# IKBKE downregulation increases chemosensitivity through pyroptosis mediated by the caspase-3/GSDME pathway in pancreatic cancer

**DOI:** 10.1186/s13046-026-03670-1

**Published:** 2026-02-16

**Authors:** Ting Ren, Xue Chen, Xiaozhen Wang, Yantian Xu, Na Shao

**Affiliations:** 1 Shandong Provincial Hospital Affiliated to Shandong First Medical University, 324 Jingwu Rd, Jinan, 250021 China; 2 Department of Oncology, Shandong Provincial Hospital Affiliated to Shandong First Medical University, 324 Jingwu Rd, Jinan, 250021 China; 3 Shandong University of Traditional Chinese Medicine, 4655 Daxue Rd, Jinan, 250355 China; 4 Department of Liver Transplantation and Hepatobiliary Surgery, Shandong Provincial Hospital Affiliated to Shandong First Medical University, 324 Jingwu Rd, Jinan, 250021 China

**Keywords:** Pancreatic ductal adenocarcinoma, IKBKE, Chemosensitivity, Pyroptosis, GSDME

## Abstract

**Background:**

The aggressive cancer known as pancreatic ductal adenocarcinoma (PDAC) has a remarkably poor response to treatment, especially gemcitabine (GEM). As a member of the noncanonical IκB kinase family, inhibitor of nuclear factor kappa-B kinase subunit epsilon (IKBKE) is known to regulate tumor progression in multiple cancer types. However, its functional role in modulating chemosensitivity and its impact on cell death pathways in PDAC remain unclear.

**Materials and methods:**

We examined IKBKE expression in patient tumor tissues and publicly available databases and assessed its prognostic value to investigate its function in PDAC chemoresistance. Loss-of-function approaches, including shRNA knockdown and pharmacological inhibition using amlexanox, were employed in vivo and in vitro. Furthermore, cytotoxicity along with cell death patterns induced by GEM were assessed through flow cytometry, electron microscopy, and CCK-8 assays. Co-IP and GST pull-down assays were applied to determine whether IKBKE interacts with gasdermin E (GSDME), the executioner protein of pyroptosis. Moreover, in vitro kinase assays, phosphorylation mass spectrometry, and site-specific gain-/loss-of-function mutant functional experiments were conducted to investigate the specific pathways via which IKBKE affects GSDME-mediated pyroptosis. The therapeutic potential of IKBKE targeting was further validated using patient-derived tumor organoids and xenograft models.

**Results:**

IKBKE was significantly elevated in PDAC tissues and was closely linked to worse clinical outcomes. Functional studies of the signaling pathway showed that via the activation of the protein kinase B/glycogen synthase kinase-3β signaling pathway, IKBKE facilitates the aggressive phenotype of PDAC cells. IKBKE inhibition or silencing increased GEM sensitivity and caused caspase-3/GSDME-dependent pyroptosis in PDAC cells. Co-IP and GST pull-down analysis revealed IKBKE–GSDME interaction. Further in vitro cleavage and kinase assays, mass spectrometry analysis, and mutant functional studies showed that IKBKE inhibits pyroptosis by phosphorylating GSDME at Thr6, thereby hindering its cleavage by caspase-3. Treatment with amlexanox markedly suppressed tumor growth and synergized with GEM to enhance anti-tumor efficacy in organoid models by targeting IKBKE.

**Conclusion:**

Our findings identify IKBKE as a key regulator of chemotherapy-induced pyroptosis in PDAC. Specifically, IKBKE phosphorylates GSDME at Thr6, which alters its conformation, thereby impeding caspase-3–mediated cleavage, ultimately contributing to GEM chemoresistance. IKBKE targeting reverses GEM resistance and enhances tumor immunogenic cell death via the caspase-3/GSDME pathway, supporting IKBKE inhibition as a promising therapeutic strategy for PDAC.

**Graphical Abstract:**

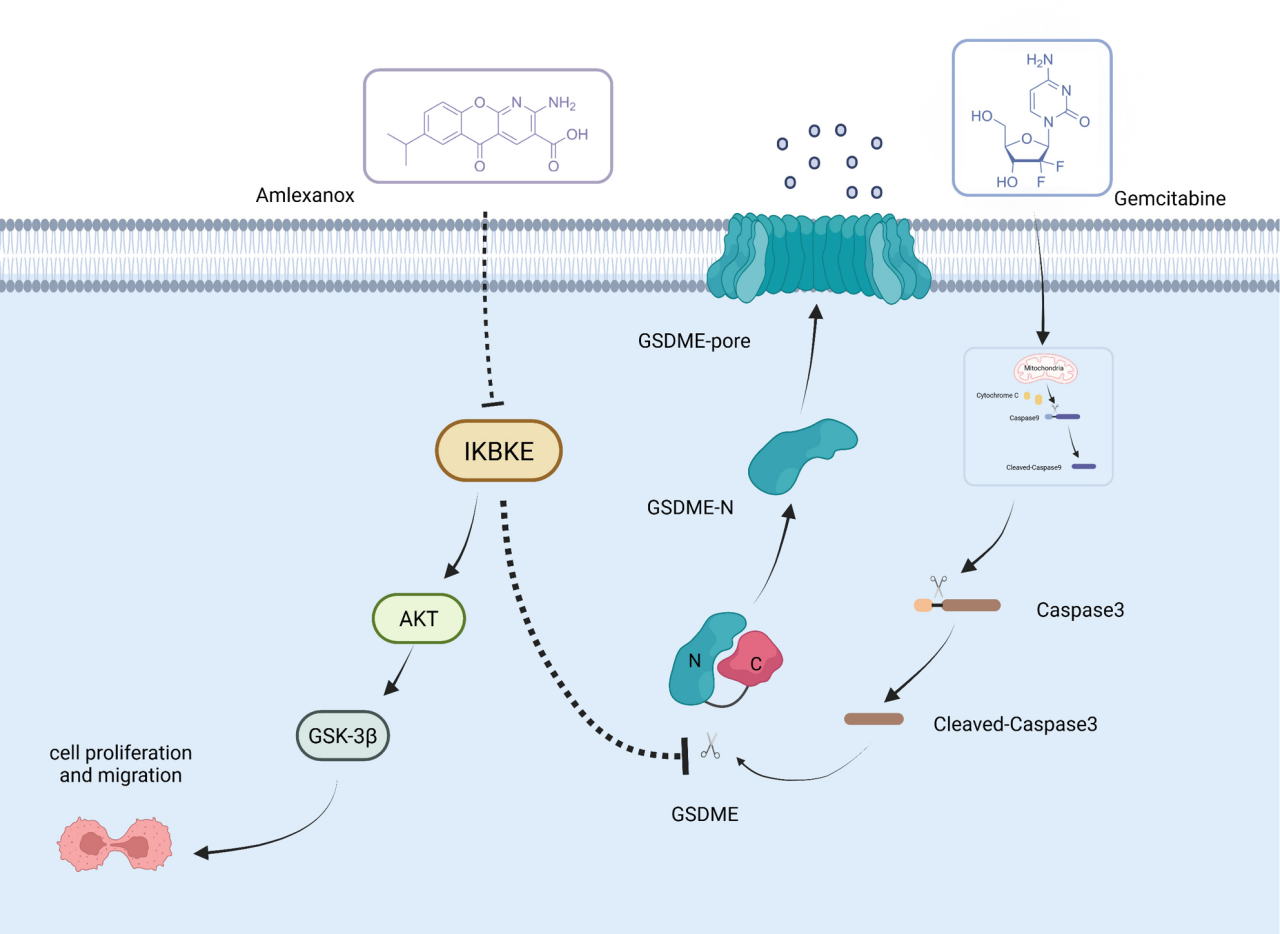

**Supplementary Information:**

The online version contains supplementary material available at 10.1186/s13046-026-03670-1.

## Introduction

About 90% of instances of pancreatic cancer are pancreatic ductal adenocarcinoma (PDAC), a malignant tumor that is common in the digestive system [1]. Recent GLOBOCAN data indicated that pancreatic cancer caused 467 000 deaths worldwide in 2022 [2, 3]. The major treatment option for locally advanced along with metastatic pancreatic cancer is GEM, either by itself or in conjunction with other treatments [4, 5]. However, survival with GEM-based chemotherapy was extended by only a few weeks to a few months [6]. Therefore, novel therapeutics aimed at enhancing chemosensitivity and improving the prognosis of patients with PDAC are warranted [7].

IKBKE, a member of the IKK family, is crucial for regulating inflammatory reactions, immune cell activation and proliferation, as well as metabolic diseases [8]. Recent studies have reported that IKBKE facilitates the growth, proliferation, and invasion of various tumors, including esophageal, glioma, breast, liver, and gastric cancers, as well as non-small cell lung cancers [9–14]. Moreover, IKBKE is involved in chemosensitivity regulation, and IKBKE inhibition increases the sensitivity of triple-negative breast cancers and glioblastoma multiforme to chemotherapeutic drugs [15, 16]. However, the mechanism underlying the association between IKBKE and chemosensitivity in pancreatic cancer needs to be elucidated.

GEM is widely utilized in PDAC treatment [17, 18]. However, chemoresistance to GEM remains a major clinical challenge [19, 20]. Iede et al. [21] found that OXCT1 overexpression markedly improved the anti-apoptotic ability of PDAC cells, thus strengthening tolerance to GEM via the NF-κB signaling pathway. According to Du et al. [22], the E3 ubiquitin ligase UBR5 induced the epithelial–mesenchymal transition mediated by O-GlcNAcylation, which led to resistance of pancreatic cancer to GEM, whereas targeting UBR5 with Y-39983 dihydrochloride reversed such a resistance. However, the mechanism underlying GEM resistance in PDAC is poorly understood, warranting further studies to develop novel therapeutic strategies to overcome this GEM resistance.

It has long been known that cytotoxic drug-induced apoptosis is a significant mechanism for cancer cell death [23]. However, targeting nonapoptotic mechanisms, such as pyroptosis, has gained attention as cancer cells often develop resistance to chemotherapy-induced apoptosis during treatment [24]. Apoptosis and pyroptosis are caspase-dependent programmed death pathways [25]. Caspase-3 is a pivotal protein that acts as a switch between pyroptosis and apoptosis [24, 26]. According to recent research, chemotherapeutic medications cause pyroptosis via the caspase-3/GSDME pathway [27]. Caspase-3/GSDME regulation can be an alternative cancer treatment strategy for increasing chemosensitivity. However, novel strategies to increase chemotherapy-induced pyroptosis and chemosensitivity require further investigation.

The carcinogenic impact of IKBKE along with its relationship to chemosensitivity in PDAC were examined in this work. Patients with PDAC were found to have high IKBKE expression. Overall survival (OS) can be independently predicted by the level of IKBKE expression. Through the protein kinase B/glycogen synthase kinase-3β (AKT/GSK-3β) pathway, IKBKE contributes to carcinogenesis. IKBKE downregulation sensitizes PDAC cells to GEM and increases GEM-induced pyroptosis via the caspase-3/GSDME pathway. Through co-immunoprecipitation (co-IP) and glutathione S-transferase (GST) pull-down assays, a protein interaction between IKBKE and GSDME was identified. Further mechanistic investigation showed that IKBKE inhibits pyroptosis by phosphorylating GSDME at threonine 6 (Thr6), thereby preventing its cleavage by caspase-3. Furthermore, using cell lines, patient-derived tumor organoids (PDOs), and animal models, amlexanox (AMX), an IKBKE inhibitor, was found to inhibit the proliferation of PDAC and enhance its sensitivity to GEM. Our results provide experimental support and a theoretical foundation for IKBKE targeting to increase chemosensitivity by stimulating pyroptosis in PDAC.

## Materials and methods

### Patients and tissue samples

This work enrolled 103 patients with PDAC and 62 with pancreatic cystadenoma who underwent radical surgery between December 1, 2015, and July 30, 2018, at Shandong Provincial Hospital Affiliated to Shandong First Medical University. The exclusion criteria were as follows: perioperative mortality, distant metastasis, lost to follow-up, and insufficient tumor tissue.

This research protocol has been authorized by the Institutional Ethics Committee of Shandong Provincial Hospital Affiliated to Shandong First Medical University (NFSC: NO.2021 − 767). The patients’ medical records were the source of all clinical data. Supplementary Table S1 presents clinicopathologic variables for all patients.

### Cell culture and lentiviral infection

CFPAC-1 and BxPC-3 cell lines were acquired from SevierBio (Wuhan, China). A human normal pancreatic duct cell line, hTERT-HPNE, was supplied by FUHENG Biotech (Shanghai, China). Prof. Xue from Shandong University kindly provided the PANC-1 and MiaPaCa-2 cell lines. Short tandem repeat profiling was performed for all cell lines.

All plasmids were confirmed through DNA sequencing. The human HA-IKBKE, Flag-GSDME (full-length), and the inducible Flag-GSDME (1–270, N-terminal fragment) expression plasmid was constructed by Boshang Biotech (Jinan, China). The inducible vector is based on the Tet-On 3G system, and its expression is induced by doxycycline. Furthermore, QuikChange site-directed mutagenesis was utilized to create Flag-GSDME (T6A and T6E) point mutant plasmids.

Lentiviral vectors carrying human IKBKE-specific shRNA (target sequences: 1: 5′-CCAGATGCTCCCAAAATATCA-3′, 2: 5′-CGGCATTGTGCATCGCGACAT-3′, 3: 5′-CAAGTTCGTCCCCAAAGTGGA-3′) and a nontargeting control (5′-AAACGTGACACGTTCGGAGAACGAATTCTCCGAACGTGTCACGTTT-3′) were obtained from SyngenTech (Beijing, China). Control and IKBKE-overexpression vectors were developed by GeneChem (Shanghai, China). Utilizing western blot analysis, the efficacy of gene knockdown or overexpression was assessed.

### CCK-8 assay

PDAC cells (1 × 10^3^ cells/well) were incubated into 96-well plates and allowed to adhere for 24 h. Following treatment with either AMX (10 µM) or GEM (20 µM), the cells were grown for 72 h. A spectrophotometer (Thermo Fisher Scientific, MA, USA) was employed to measure the sample’s absorbance at 450 nm.

### EdU incorporation assay

PDAC cells (1 × 10^4^ cells/well) were either treated with AMX (10 µM) in 24-well plates or left untreated. Following a 24-hour period, the DNA replication rate was measured utilizing the Cell-Light™ EdU Imaging Detection Kit (Ruibo Biotechnology, Guangzhou, China) in accordance with the manufacturer’s instructions. A fluorescent microscope (Carl Zeiss AG, Oberkochen, Germany) was applied to get representative images.

### Colony formation assay

PDAC cells (1 × 10^3^ cells/well) were injected onto 6-well plates and left to adhere for 24 h. Following treatment with AMX (10 µM), LY 294002 (10 µM), or 9-ING-41 (10 µM), the cells were incubated for two weeks, with medium changes occurring every 3–4 days. Colonies containing more than fifty cells were deemed positive.

### 12-well plate staining

PDAC cells (1 × 10^4^ cells/well) were injected into 12-well plates and left to adhere for one day. The cells were either treated with 20 µM GEM in addition to necrostatin-1 (Nec, 20 µM), ferrostatin-1 (Fer, 10 µM), chloroquine (Chlo, 30 µM), MCC950 (MCC, 10 µM), disulfiram (Dis, 1 µM), Z-DEVD-FMK (DEVD, 10 µM) or Z-VAD-FMK (VAD, 10 µM) or left untreated, and then were cultivated for 72 h, fixed with 4% paraformaldehyde, and finally stained utilizing 0.5% crystal violet.

### Transwell assay

Complete medium (600 µL) that contains 10% FBS was incorporated to the lower chamber of a 24-well Transwell plate. Serum-free medium (200 µL) was utilized to resuspend PDAC cells (1 × 10^3^ cells/well). Subsequently, the cells were transferred to the upper chamber. After 6 h, 9-ING-41 (10 µM), LY 294002 (10 µM), or AMX (10 µM) was introduced into the upper chambers and cultivated for another 24 h. Crystal violet staining was performed on migrating cells, and an optical microscope was employed to capture representative images.

### Wound-healing assay

In 6-well plates, 1 × 10^4^ PDAC cells were cultured in each well. As the cells achieved 70%–80% confluence, medium with AMX (10 µM) was added and left for 2 h, after which scratches were made using a sterile pipette tip. To evaluate cell migration, at 0, 24, and 48 h, the scratches were detected and photographed using an optical microscope.

### Live/dead cell assay

PDAC cells (1 × 10^4^ cells/well) were seeded into 24-well plates and cultivated for 12 h. For 24 h, the cells were either treated with GEM (20 µM) in addition to AMX (10 µM), LY-294002 (10 µM), or 9-ING-41 (10 µM) or left untreated and then stained using the Calcein-AM/EthD-I Live/Dead Cell Double Staining Kit (CA1631, Solarbio, Beijing, China). Under a fluorescence microscope (Carl Zeiss AG), the stained cells were observed, with live and dead cells fluorescing green and red, respectively. Cell counts were quantified using ImageJ.

### Electron microscopy detection

PDAC cells (5 × 10^6^ cells/well) were seeded in 75-cm² flasks and processed by GEM (20 µM) for 72 h. Cells were then harvested and fixed with 2.5% glutaraldehyde (Solarbio) for 24 h, followed by dehydration, embedding, and staining utilizing uranyl acetate and lead citrate. Subsequently, the samples were examined with a TEM (JEM-1400PLUS; JEOL Ltd., Japan).

### Flow cytometry

GEM (20 µM) was administered for 72 h after PDAC cells (1 × 10^4^ cells/well) were injected onto 6-well plates. The cells were gathered using trypsin free of ethylenediaminetetraacetic acid, cleaned using PBS, and stained via the Annexin V-FITC/PI Assay Kit. The stained cells were examined with the FACS Celesta**™** flow cytometer (Becton, Dickinson and Company, USA).

### LDH release assay

GEM (20 µM) was used to culture PDAC cells (1 × 10^3^ cells/well) for 24 h after they were seeded into 96-well plates. The LDH Cytotoxicity Assay Kit (Beyotime, Shanghai, China) was exploited to measure lactate dehydrogenase (LDH) release.

### PDO culture

From February to July 2024, seven PDAC tissue samples were collected, and four PDOs were successfully established (success rate, 57.14%). PDAC specimens were minced and digested in a tissue digestion medium (Biogenous, Jiangsu, China) in a water bath for 1–2 h. The digested specimens were neutralized by adding PBS containing 1% BSA and centrifuged at 3 000 ×g. Then, after the cells were resuspended in ice-cold Geltrex Matrigel (Corning, NY, USA), 50 µL droplets of Matrigel/cell solution were planted onto 24-well plates. By inverting the culture dish, the Matrigel/cell suspension was allowed to settle for 30 min in the cell culture incubator. Subsequently, a pancreatic cancer-specific medium (250 µL) with 2.5 µL of Y-27632 (10 µM, Cayman Chemical, Ann Arbor, MI, USA) was included in each well, with the medium replaced every 3 days. An optical microscope (Carl Zeiss AG) was used to obtain representative images.

### PDO drug responsiveness assay

Each well of a black 96-well plate (Corning) was filled with 1.5 µL of Matrigel/PDO suspension. When the diameter of the PDOs reached 100 μm, they were co-cultured with GEM (20 µM) and/or AMX (10 µM) for 3 days. Representative images were taken using an optical microscope (Carl Zeiss AG). Subsequently, introduce 100 µL of CellTiter-Lumi™ Luminometer Cell Viability Assay (Beyotime) into each well. A microtiter plate was incubated for 30 min at room temperature after after gentle shaking. Afterwards, a SpectraMax Mini multimode microplate reader (BIOGORGE, Shanghai, China) was exploited to determine the luminescence.

### Human phosphokinase array

The relative levels of 39 phosphorylated proteins were evaluated using the Human Phospho-Kinase Array Kit (R&D Systems, USA). Phosphatase and protease inhibitors were added to RIPA buffer, which was then used to lyse the cells. Protein lysates were incubated with array membranes. Amersham Imager 680 (GE AI, USA) was utilized for signal detection, and the results were analyzed using ImageJ.

### Western blot

Tissues and cells were lysed on ice utilizing RIPA buffer (Solarbio), which contains phosphatase and protease inhibitors. The concentrations of protein were determined via the Bicinchoninic Protein Assay Kit (Beyotime). Enhanced chemiluminescence was utilized to visualize the proteins following western blot analysis. The primary antibodies utilized were anti-IKBKE (1:1 000, CST, 2905), anti-IKBKE (1:1 000, HUABIO, ET1706-20), anti-IKBKE (1:1 000, Proteintech, 68531-1-Ig), anti-GSDME (1:1 000, Abcam, ab215191), anti-GSDME (1:2 000, Proteintech, 67731-1-Ig), anti-GSDMB (1:1 000, CST, 76439), anti-GSDMD (1:1 000, CST, 39754), anti-caspase-3 (1:1 000, CST, 9662), anti-p-AKT T308 (1:1 000, CST, 9275), anti-p-AKT S473 (1:1 000, CST, 9271), anti-AKT (1:1 000, CST, 9272), anti-p-GSK-3β (S9) (1:1 000, CST, 9336), anti-GSK-3β (1:1 000, CST, 9315), anti-vinculin (1:1 000, CST, 4650), and anti-GAPDH (1:10 000, Proteintech, 10494-1-AP). Goat-anti-rabbit secondary antibody was used at 1:5 000 dilution (Zhongshan Biotechnology, Beijing, China).

### Co-IP

PDAC cells were extracted at 90% confluence after being plated in 75-cm² flasks. A lysis solution with protease and phosphatase inhibitors was applied to lyse the cells. The lysates were precleared with anti-IgG antibodies and centrifuged to exclude nonspecifically bound proteins. Then, specific antibodies were added to the lysates to precipitate the target proteins and their interacting partners. Following treatment with protein A/G agarose beads, the protein-antibody complexes were purified by centrifugation. Finally, the target proteins and their interacting partners were eluted and detected via western blot analysis.

### GST pull-down assay

The procedure was carried out utilizing the Pierce™ GST Protein Interaction Pull-Down Kit (21516, Thermo Fisher) and purified His-GSDME and GST-IKBKE proteins.

### In vitro kinase assays

As previously mentioned, kinase reactions were implemented [28]. In 40 µL kinase buffer (25 mM Tris-HCl, pH 7.5), 2 mM DTT, 5 mM beta-glycerophosphate, 10 mM MgCl_2_ and 0.1 mM Na_3_VO_4_, purified GST-IKBKE (100 ng) was inoculated with purified His-GSDME (500 ng) and 50 µM ATP-γ-S (A274887, Aladdin) at 30 °C for 60 min. Samples that had been treated with ATP-γ-S were alkylated for 1 h at room temperature using 5 mM p-nitrobenzyl mesylate in 5% dimethyl sulfoxide. SDS-PAGE loading buffer was incorporated, and the processes were terminated by heating them to 95 °C for 10 min. The reaction mixtures were then analyzed using immunoblotting. The tagged substrates were identified with an anti-thiophosphate ester antibody (HUABIO, ET1612-11).

### In vitro cleavage assay

Active caspase-3 (100 ng) was incubated with either mutant or wild-type (WT) GSDME (500 ng) in a 20 µL reaction buffer containing 100 mM NaCl, 50 mM HEPES (pH 7.5), 10 mM DTT, 0.1% CHAPS, 10% glycerol, and 1 mM EDTA. After adding 10 µL of 2× SDS loading buffer and heating to 95 °C for 10 min, the reaction was terminated.

### Mass spectrometry analysis

Purified GST-IKBKE protein was used to produce phosphorylated His-GSDME samples, as explained in the section describing the in vitro kinase test. Following SDS-PAGE, the phosphorylated His-GSDME protein (5 µg) was stained with Coomassie blue (P1300, Solarbio). Thereafter, the specific protein band was isolated, treated with trypsin digestion, and subsequently detected via mass spectrometry.

### In vivo experiment

The Shandong Provincial Ethics Committee (No.2023 − 174) authorized all animal studies, which were implemented following the guidelines of Shandong Provincial Animal Model Research Institute. Four-week-old female Balb/c nude mice were supplied by Jinan Pengyue Experimental Animal Breeding (Shandong, China). Tumor dimensions were recorded every three days, and volumes were calculated using the formula: tumor volume (mm^3^) = (ab^2^)/2, in which, a and b denote the long and short diameters, separately. The tumors were paraffin-embedded, with 4% paraformaldehyde treatment, and stained with antibodies after the mice were euthanized after three weeks.

- Experiment 1: Mice were randomly divided into three groups (n = 7 per group): (1) IKBKE-NC, (2) OE-IKBKE, and (3) OE-IKBKE + AMX. NC or IKBKE-overexpressing PANC-1 cells (1 × 10^7^) were injected subcutaneously into the axilla of mice. One week after cell inoculation, the OE-IKBKE + AMX group administered intraperitoneal injections of AMX (30 mg/kg) every 3 days for 2 weeks.
- Experiment 2: Mice were randomly assigned to four groups (n = 7 each group): (1) NC, (2) shIKBKE, (3) NC + GEM, and (4) shIKBKE + GEM. IKBKE-knockdown CFPAC-1 cells (1 × 10⁷) were subcutaneously injected into the axilla of mice. One week after cell inoculation, the NC + GEM and shIKBKE + GEM groups received intraperitoneal injections of GEM (50 mg/kg) every 3 days for 2 weeks.

### IHC and staining analysis

PDOs (up to 200 μm in diameter) and tissues were fixed in 4% paraformaldehyde solution for 24 h, followed by paraffin embedding and sectioning into 4 μm slices. The tissue slices were deparaffinized and rehydrated following a 2-hour incubation period at 65 °C. The sections were heated to 95 °C in 0.01 M citrate buffer. Furthermore, the tissue was blocked using normal goat serum. The tissues were treated via a primary antibody at 4 °C overnight. The primary antibodies included anti-IKBKE (HUABIO, ET1706-20), anti-Ki-67 (Beyotime, AF1738), anti-CK7 (Proteintech, 66483-1-Ig), anti-CDX-2 (Proteintech, 60005-1-Ig), and anti-CK20 (Proteintech, 60183-1-Ig). The sections were then stained utilizing 3,3′-diaminobenzidine after being incubated with a secondary antibody for 1 h (Zhongshan Biotechnology). Following hematoxylin counterstaining, all sections were sealed and dried.

### RNA isolation and RT-qPCR

Isolation of total RNA from the cells was conducted with the RNA kit (Invitrogen, CA, USA). After that, an M-MLV reverse transcriptase (Vazyme, Nanjing, China) was used to reverse-transcribe 2 µg of RNA into cDNA. RT-qPCR was performed using the QuantStudio 3 Flex Real-Time PCR system (Applied Biosystems, USA) and SYBR Green Master Mix. The primer sequences for β-actin, GSDME, and IKBKE were as below:

- β-actin primers
  - Forward: 5′-CACCATTGGCAATGAGCGGTTC-3′
  - Reverse: 5′-AGGTCTTTGCGGATGTCCACGT-3′
- GSDME primers
  - Forward: 5′-GATCTCTGAGCACATGCAGGTC-3′
  - Reverse: 5′-GTTGGAGTCCTTGGTGACATTCC-3′
- IKBKE primers
  - Forward: 5′-GGCTACAACGAGGAGCAGATTC-3′
  - Reverse: 5′-GGACGCTTGATACTTCTGCACG-3′

### 3D structural modeling of IKBKE and caspase-3/GSDME

The 3D protein structures of GSDME and IKBKE were sourced from the AlphaFold Protein Structure Database (IKBKE: https://alphafold.ebi.ac.uk/entry/Q14164, GSDME: https://alphafold.ebi.ac.uk/entry/O60443), whereas that of caspase-3 was acquired from the PDB Protein Structure Database (PDB ID 2J31). The binding free energy and the most important residues/interactions of the protein complexes were predicted using Zdock, and the molecular structures were visualized using PyMOL.

### Statistical analysis

Data were analyzed using IBM SPSS Statistics 27.0.1 (IBM Corp., Armonk, NY, USA) and GraphPad Prism 9.5. Kaplan-Meier survival analysis was performed using the log-rank test. For additional statistical analyses, Pearson’s correlation test, ANOVA, or two-tailed Student’s t-test was used. Each experiment was independently repeated at least three times. The data were presented as mean ± SD. *P < 0.05, **P < 0.01, and ***P < 0.001 were considered statistically significant.

## Results

### IKBKE is highly expressed and associated with poor prognosis in patients with PDAC

**Fig. 1:**
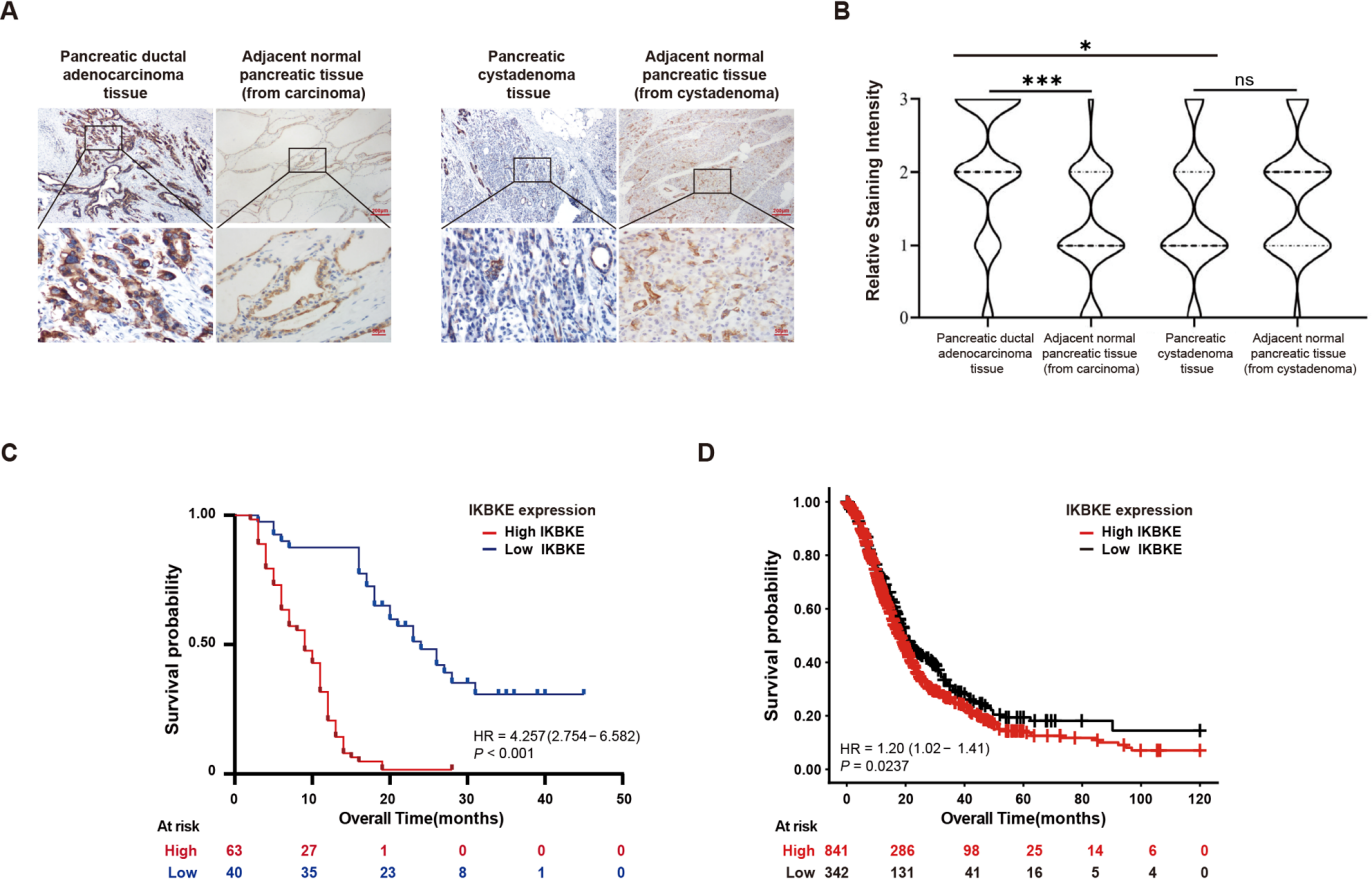
IKBKE is highly expressed and associated with poor prognosis in patients with PDAC. **A** IHC images of IKBKE staining in biopsy specimens from patients with PDAC and pancreatic cytomegaloma; scale bar = 200 μm and 50 μm, respectively. **B** Distribution of the relative staining intensity of PDAC and pancreatic cystadenoma. **C** Kaplan–Meier curves for the overall survival of patients with high (*n* = 63) and low (*n* = 40) IKBKE expression. **D** Kaplan–Meier curves of survival data in patients with different IKBKE expression in the GEO database (*n* = 1 183). *P < 0.05, **P < 0.01, ***P < 0.001.

The clinical significance of IKBKE was assessed by examining its expression in the postoperative pathological specimens of 103 and 62 patients with PDAC and pancreatic cystadenoma, respectively, via immunohistochemistry (IHC). IKBKE was more highly expressed in patients with PDAC than in those with paracarcinoma and pancreatic cystadenoma (Fig. 1A and B). PDAC patients were categorized into two groups in accordance with levels of IKBKE expression: a low IKBKE expression group (IHC scores < 2, *n* = 40) and a high IKBKE expression group (IHC scores ≥ 2, *n* = 63). According to the further survival analysis, the low IKBKE expression group revealed significantly greater OS when in contrast to the high IKBKE expression group (HR = 4.257, 95% CI: [2.754–6.582], *P* < 0.001) (Fig. 1C). The prognostic value of IKBKE was confirmed with the Kaplan–Meier Plotter online tool (https://pancreas.kmplot.com/). A greater OS was also linked to reduced IKBKE expression in pancreatic cancer patients (*n* = 1 183, HR = 1.20, 95% CI: [1.02–1.41], *P* = 0.0237) (Fig. 1D). Moreover, the IKBKE expression level showed a positive correlation with AJCC (8th edition) staging (*P* < 0.001) and T-stage (*P* < 0.001) (Table 1). The IKBKE expression level (*P* < 0.001) and T-stage (*P* < 0.001) were found to be independent predictive markers of OS by univariate and multivariate Cox regression analysis (Table 2).

**Table 1:** The correlation of clinicopathological characteristics of PDAC patients with IKBKE expression

Clinicopathological features	IKBKE expression		*P* value
	Low (*n* = 40)	High (*n* = 63)	
Age			
< 59	22	28	0.296
≥ 59	18	35	
Gender			
Female	14	20	0.732
Male	26	43	
Location			
Head of pancreas	20	27	0.478
Body and tail of pancreas	20	36	
Histologic grade			
G1	3	5	0.607
G2	20	37	
G3	17	21	
T stage			
T1	23	4	**< 0.001**
T2	12	30	
T3	5	21	
T4	0	8	
N stage			
N0	27	36	0.444
N1	12	22	
N2	1	5	
AJCC stage			
Ⅰ	31	21	**< 0.001**
Ⅱ	9	34	
Ⅲ	0	8	
Short-term death (≤ 6 months)			
Yes	4	23	**0.003**
No	36	40	
Adjuvant therapy			
Yes	7	6	0.235
No	33	57	
Hypertension			
Yes	9	16	0.738
No	31	47	
Diabetes			
Yes	9	16	0.738
No	31	47	
Coronary heart disease			
Yes	2	3	> 0.999
No	38	60	
Second primary tumor			
Yes	2	1	0.558
No	38	62	
Comorbidity (hypertension, diabetes, coronary heart disease, second primary tumor)			
Yes	16	25	> 0.999
No	24	38	

*P* value calculated by the χ^2^ test or Fisher’s exact test;

Values in bold signify *P* < 0.05, which is considered significant

*T* tumor, *N* lymph node metastasis

**Table 2:** Univariate and multivariate analyses of prognostic factors for OS in PDAC patients

Variable	Univariate analyses	OS		
		Multivariate analyses		
	*P* value	*P* value	HR	95% CI
Age	0.893			
Gender	**0.195**	0.143		
Location	0.465			
Histologic grade	0.832			
T stage	**< 0.001**	**< 0.001**	**3.978**	**2.782–5.688**
N stage	0.873			
AJCC stage	0.769			
IKBKE expression (low vs. high)	**< 0.001**	**< 0.001**	**4.072**	**2.113–7.846**
Adjuvant therapy	**0.051**	0.246		
Hypertension	0.821			
Diabetes	0.855			
Coronary heart disease	0.736			
Second primary tumor	**0.050**	0.080		
Comorbidity	0.521			

Values in bold signify *P* < 0.05, which is considered significant

*CI* confidence interval, *HR* hazard ratio, *OS* overall survival, *T* tumor, *N* lymph node metastasis

### IKBKE facilitates the malignant biological behavior of PDAC both in vitro and in vivo

**Fig. 2:**
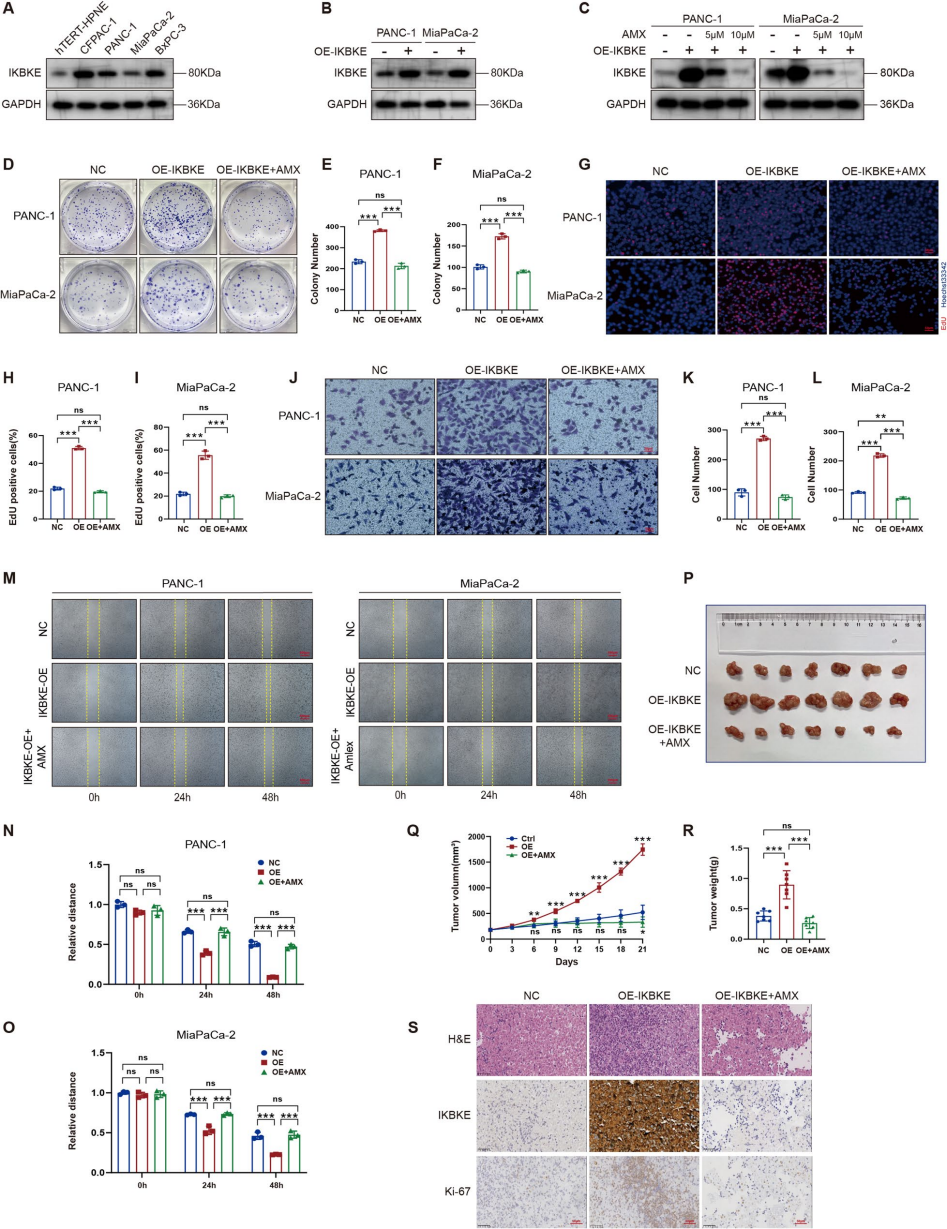
IKBKE facilitates the malignant biological behavior of PDAC both in vitro and in vivo. **A** IKBKE expression in hTERT-HPNE, PANC-1, MiaPaca-2, BxPC-3, and CFPAC-1 cells. **B** IKBKE expression in stable cell lines transfected with lentivirus encoding IKBKE (OE-IKBKE). **C** IKBKE expression after treatment with AMX in OE-IKBKE cells. **D-F** Colony formation assay of IKBKE-overexpressing cells (*n* = 3). Representative images (**D**) and quantitative analysis of colony numbers (**E, F**) are shown. **G-I** Effect of IKBKE on DNA synthesis in IKBKE-overexpressing cells (*n* = 3). Cells were fluorescently stained with EdU (red). The nucleus was stained with Hoechst 33342 (blue); scale bar = 50 μm. Representative images (**G**) and quantification of the EdU-positive cells (**H, I**) are shown. **J-L **Transwell migration of IKBKE-overexpressing cells. (**J**) Representative images; scale bar = 50 μm. (**K, L**) Quantification of migrated cells per field (*n* = 3). **M-O** Wound healing assay of IKBKE-overexpressing cells (*n* = 3); scale bar = 500 μm. Representative pictures (**M**) and quantifications (**N, O**) are shown. **P-R** Photos of the excised tumors from xenograft tumor models (**P**). NC (*n* = 7), OE-IKBKE (*n* = 7), and OE-IKBKE + AMX (*n* = 7). Tumor volumes (**Q**) and weights (**R**) were measured. **S** Representative images of H&E staining of xenograft tumor sections and IHC staining assay for IKBKE and Ki-67; scale bar = 50 μm. Mean ± SD. *P < 0.05, **P < 0.01, ***P < 0.001.

The baseline levels of IKBKE expression in normal pancreatic epithelial cells and PDAC cell lines are presented in Fig. 2A. To further explore the biological functions of IKBKE, stable cell lines were established for IKBKE overexpression (PANC-1 OE and MiaPaca-2 OE) and knockdown (BxPC-3 shIKBKE and CFPAC-1 shIKBKE) (Fig. 2B and Supplementary Fig. S1A). Previous studies have reported that AMX can inhibit IKBKE expression in glioblastoma at high concentrations [29]. A similar phenomenon was observed in PDAC cells. Western blot analysis revealed that IKBKE expression was considerably suppressed at an AMX concentration of 10 µM (Fig. 2C), and such a concentration was applied in the subsequent experiments. To assess the impact of IKBKE on PDAC proliferation, a number of tests were carried out. IKBKE upregulation enhanced PDAC cell colony size, which could be reversed by AMX, according to the colony formation test (Fig. 2D–F). Contrarily, IKBKE downregulation markedly inhibited the colony number of PDAC cells (Fig. S1B–S1D). EdU staining revealed that IKBKE-overexpressing cells exhibited a greater DNA replication rate in contrast to the control cells, which could likewise be reversed by AMX (Fig. 2G–I). Moreover, IKBKE knockdown reduced the DNA replication rates (Fig. S1E–S1G). Transwell and wound healing assays showed that IKBKE overexpression increased the migratory capacity of PDAC cells, while IKBKE knockdown or AMX treatment reduced it (Fig. 2J–O and S1H–S1M). Furthermore, cell-derived xenografts (CDXs) models were established (Fig. S1N). The results indicated that IKBKE overexpression promoted xenograft growth, resulting in an increase in tumor volume and weight (Fig. 2P–R), whereas AMX treatment suppressed xenograft growth (*P* < 0.001). IHC staining indicated that the expression of Ki-67, a marker of cell proliferation, was markedly elevated in IKBKE-overexpressing cells (Fig. 2S). Conversely, Ki-67 and IKBKE expressions significantly decreased after AMX treatment. Pathological examinations of the heart, liver, kidney, and lung tissues of nude mice treated with AMX did not reveal any abnormalities (Fig. S1O), suggesting that the inhibitory effects of AMX are at a safe concentration. The above results collectively indicate that IKBKE promotes PDAC cell proliferation in vivo.

### IKBKE promotes the malignant biological behavior of PDAC cells via the AKT/GSK-3β pathway

**Fig. 3:**
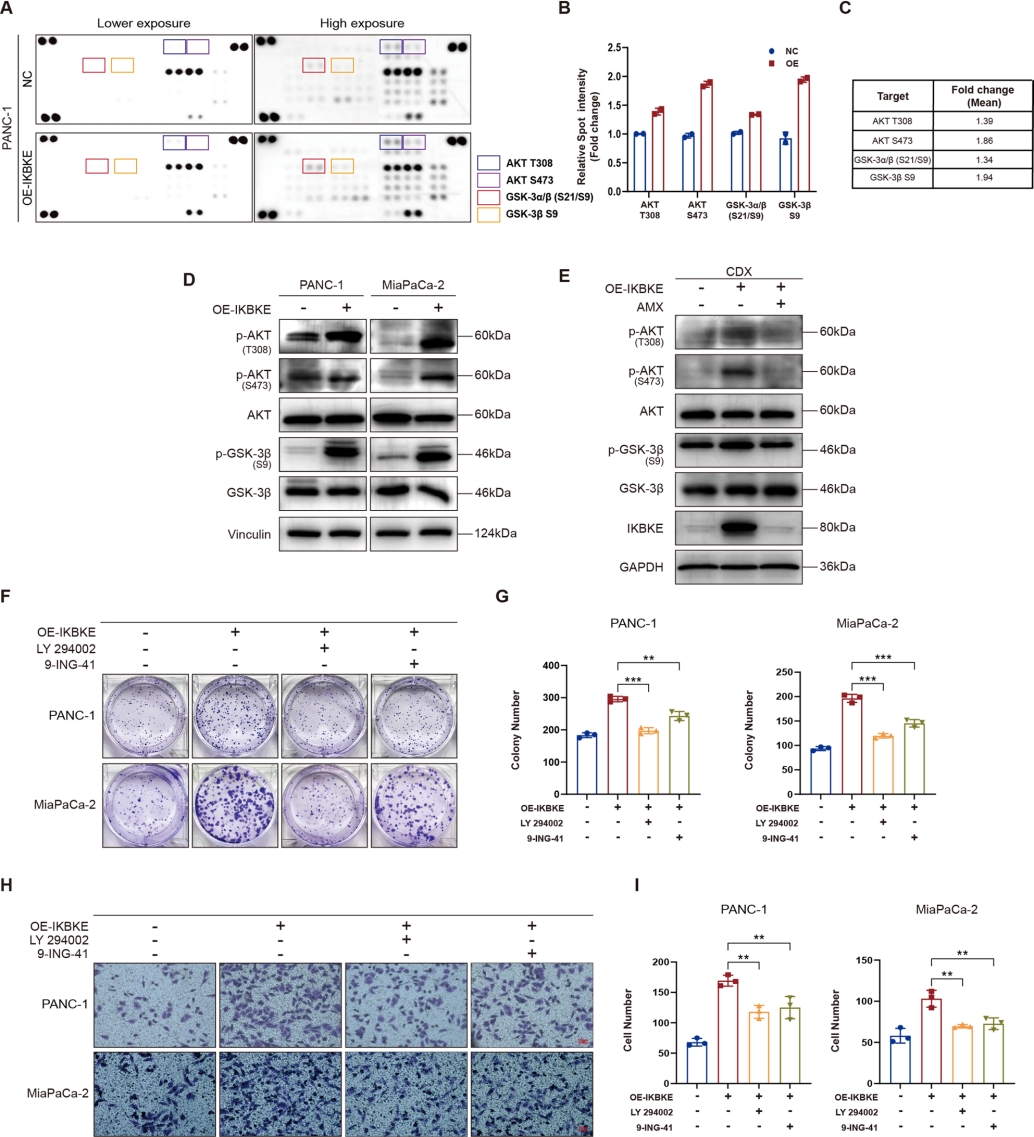
IKBKE promotes the malignant biological behavior of PDAC cells via the AKT/GSK-3β pathway. **A** Phospho-kinase array analysis in PANC-1 NC and IKBKE-overexpressing cells. **B, C** Spot intensity fold changes (*n* = 2) for individual phosphorylation of kinases normalized to control spots in PANC-1 NC cells relative to IKBKE-overexpressing cells presented as a bar chart (**B**) and tabulated (**C**). **D, E** Proteins analyzed in NC and IKBKE-overexpressing cells (**D**) and in xenograft tumors (**E**). **F,** **G** Colony formation assay in IKBKE-overexpressing cells treated with LY294002 or 9-ING-41 (*n* = 3). Representative images (**F**) and quantification of colony number (**G**) are shown. **H, I** Transwell assay of IKBKE-overexpressing cells treated with LY294002 or 9-ING-41. Representative images (**H**; scale bar = 50 μm) and quantification of the cell number (**I**, *n* = 3) are shown. Mean ± SD. *P < 0.05, **P < 0.01, ***P < 0.001.

A human phosphokinase array was employed to assess the variations in the expression of 39 phosphorylated proteins so as to further investigate the possible mechanisms through which IKBKE exerts its biological functions in PDAC. The findings indicated that the IKBKE-overexpressing group had considerably higher levels of AKT phosphorylation at S473 and T308 and GSK-3β phosphorylation at S9 (Fig. 3A–C). The GSK-3β and AKT phosphorylation levels of both xenografts (Fig. 3E) and PDAC cells (Fig. 3D) were assessed. IKBKE overexpression markedly enhanced AKT and GSK-3β phosphorylation levels, an effect that might be blocked by AMX, confirming AKT/GSK-3β pathway activation. To examine the association between the activation of AKT/GSK-3β pathway and malignant phenotype enhancement resulting from IKBKE overexpression, the malignant PDAC cell phenotype was reevaluated after pathway inhibition. The results indicated that LY294002 (an AKT phosphorylation inhibitor) and 9-ING-41 (a GSK-3β phosphorylation inhibitor) effectively inhibited the proliferative (Fig. 3F and G) and migratory ability (Fig. 3H and I) of PDAC cells, suggesting that IKBKE can promote PDAC tumorigenesis through the AKT/GSK-3β pathway.

### IKBKE affects PDAC sensitivity to GEM

**Fig. 4:**
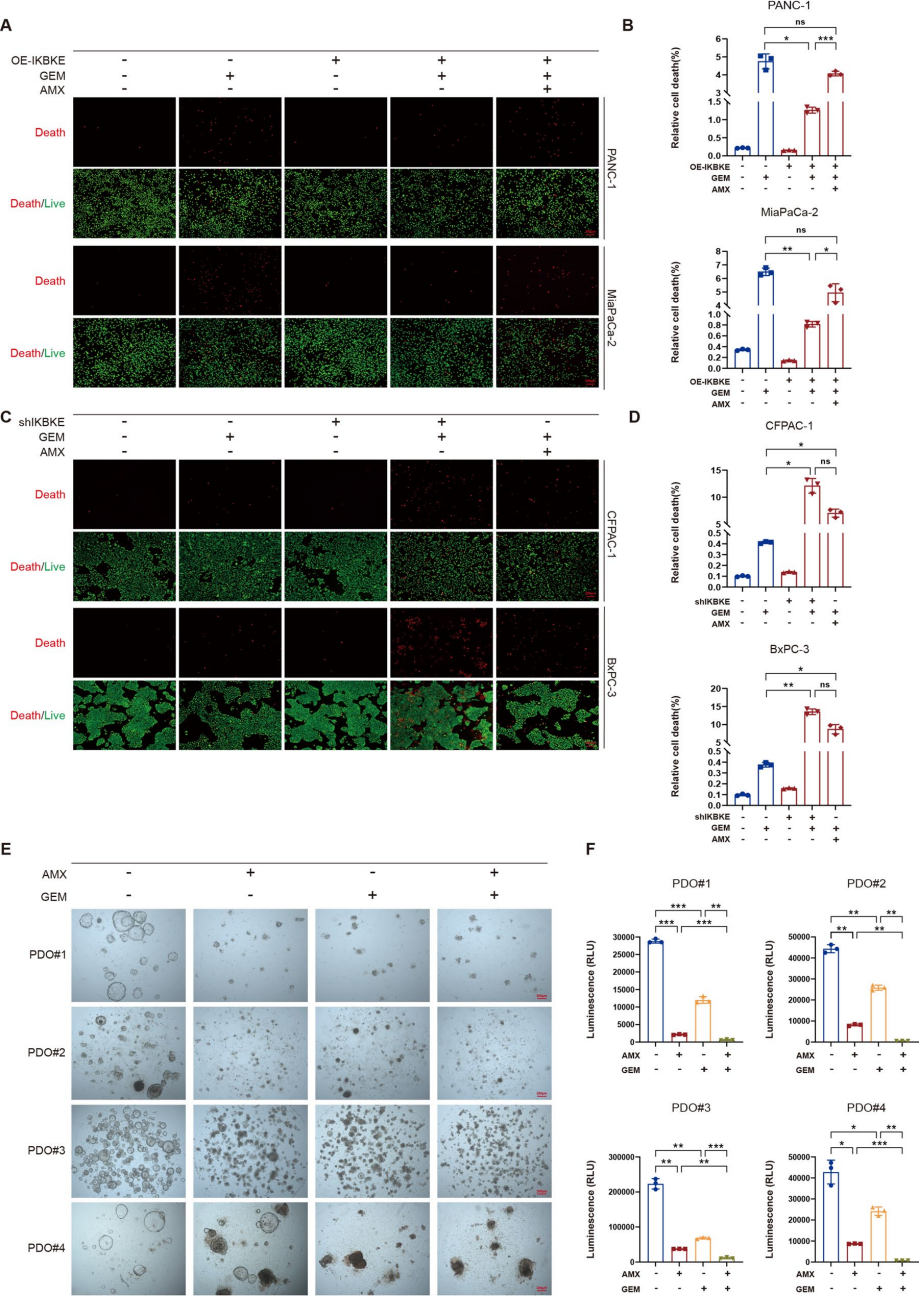
IKBKE affects PDAC sensitivity to GEM. **A-D** Live/dead cell assay (*n* = 3) showing the dead (red) and live (green) cells among IKBKE-overexpressing (**A**) and IKBKE-knockdown (**C**) cells treated with GEM or/and AMX; scale bar = 200 μm. The quantification of the relative cell death is shown (**B, D**). **E** Representative images of PDOs treated with GEM and/or AMX; scale bar = 200 μm. **F** CellTiter-Lumi™ luminescent cell viability assay detection of PDO viability (*n* = 3). Mean ± SD. *P < 0.05, **P < 0.01, ***P < 0.001.

IKBKE promotes the malignant behavior of PDAC, prompting further investigation into its function in PDAC treatment. As GEM is a first-line chemotherapeutic agent for PDAC [18], this study aimed to investigate how IKBKE affects GEM sensitivity. First, the impact of IKBKE on PDAC cell susceptibility to GEM was examined. The results indicated that IC_50_ values for GEM were notably elevated in PDAC cells with IKBKE overexpression, while its knockdown reduced IC_50_ values (Table 3). To assess the impact of IKBKE on GEM-induced PDAC cell death, live/dead cell staining assays were performed. The findings indicated that GEM-induced cell death was reduced in the IKBKE-overexpressing group in contrast to the control group, and this effect could be reversed effectively through AMX treatment (Fig. 4A and B). Furthermore, both genetic knockdown and pharmacological inhibition of IKBKE markedly enhanced GEM sensitivity (Fig. 4C and D). These results indicate that IKBKE expression influences PDAC cell sensitivity to GEM.

**Table 3:** Half-maximal inhibitive concentration (IC_50_) values of IKBKE-overexpressing and -knockdown cell lines treated GEM for 72 h

Cell lines IC_50_ 72 h (µM) [95% CI (µM)]	IKBKE-overexpressing		IKBKE-knockdown	
	PANC-1	MiaPaCa-2	CFPAC-1	BxPC-3
NC	11.88 [10.51 to 13.41]	23.20 [19.12 to 28.55]	27.47 [23.76 to 32.13]	3.089 [2.467 to 3.829]
OE-IKBKE/ shIKBKE	23.38 [19.79 to 27.8]	71.32 [62.77 to 82.28]	16.44 [13.94 to 19.57]	1.819 [1.395 to 2.314]

PDO models provide a more accurate representation of patients’ drug responsiveness compared with cellular models [30]. Therefore, PDOs were used to validate these findings. As presented in Fig. S2C, four PDO models were established to investigate the impact of IKBKE on the sensitivity of PDAC to GEM. Hematoxylin and eosin staining revealed patient-specific heterogeneous morphologies in the PDOs, whereas IHC revealed consistent expression patterns of Ki-67, CK7, CDX-2, and CK20 in both PDOs and their corresponding pathological specimens (Fig. S2D). Moreover, the IHC results confirmed IKBKE expression in all four PDOs. To examine the viability of PDOs in the treatment groups, the CellTiter-Lumi™ Luminescent cell viability assay was employed to assess PDO survival (Fig. 4E and F). The findings revealed that the combined application of AMX and GEM significantly lowered the survival rate of PDOs and that pharmacological IKBKE inhibition significantly enhanced PDO sensitivity to GEM.

Collectively, data from cell and PDO models indicated that IKBKE affects PDAC sensitivity to GEM and may be a potential target for increasing efficacy. However, the specific mechanisms involved warrant further investigation.

### IKBKE downregulation enhances sensitivity to GEM via induction of pyroptosis

**Fig. 5:**
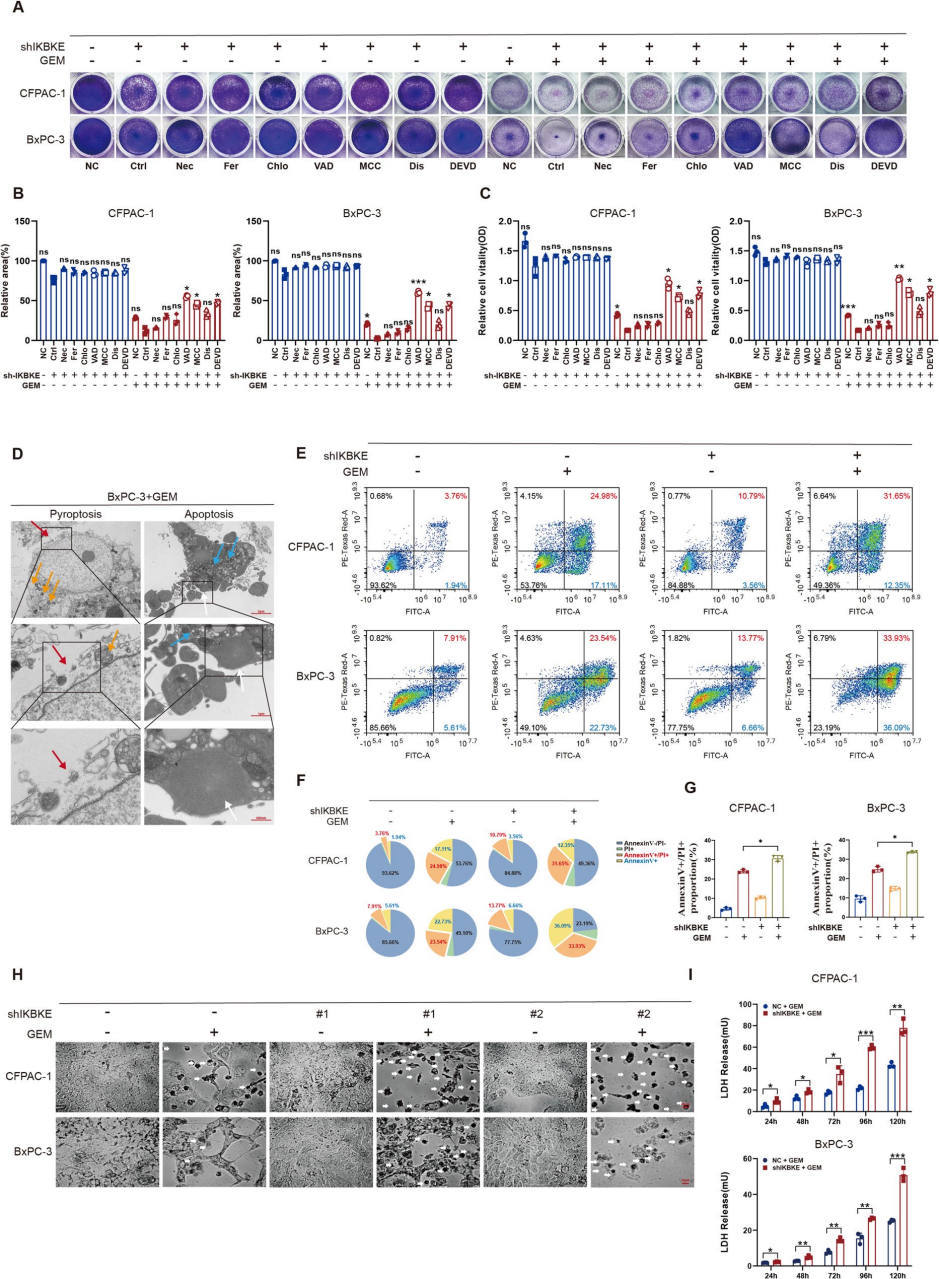
IKBKE downregulation enhances sensitivity to GEM via induction of pyroptosis. **A-C** Inhibition of distinct cell death pathways in IKBKE-knockdown cells following GEM treatment (*n* = 3). (**A**) Representative images of twelve-well plate staining. (**B**) Quantification of the stained area. (**C**) Cell viability assessed under corresponding conditions. Pharmacological inhibitors used include: necrostatin-1 (Nec), ferrostatin-1 (Fer), chloroquine (Chlo), Z-VAD-FMK (VAD), MCC950 (MCC), disulfiram (Dis), and Z-DEVD-FMK (DEVD). **D** Representative transmission electron microscopy images of pyroptosis and apoptosis in BxPC-3 cells treated with GEM; scale bars = 5 μm, 1 μm and 500 nm, respectively. Arrows denote key ultrastructural features: red, plasma membrane pores on pyroptotic cells; yellow, swollen organelles; white, characteristic apoptotic bodies; blue, condensed organelles. **E-G** Flow cytometry analysis of IKBKE-knockdown cells treated with GEM and stained with Annexin V-FITC/PI (**E**, *n* = 3). The percentage of pyroptotic cells (Annexin V+, PI+) was labeled red, and the percentage of apoptotic cells (Annexin V+, PI−) was labeled in blue. The data are displayed in a pie chart (**F**) and a bar chart (**G**). **H** Representative optical microscope images of IKBKE-knockdown cells treated with GEM. White arrows indicate pyroptotic cells with characteristic bubble-like protrusions; scale bar = 20 μm. **I** LDH release in IKBKE-knockdown cells treated with GEM (*n* = 3). Mean ± SD. *P < 0.05, **P < 0.01, ***P < 0.001.

Our previous data demonstrated that AKT/GSK-3β pathway activation is essential for IKBKE to exert its protumor effects. We next sought to determine how IKBKE modulates GEM sensitivity. We first examined whether reduced sensitivity to GEM induced by IKBKE is linked to the activation of this pathway. The results of live/dead cell staining indicated that the inclusion of pathway inhibitors did not increase the incidence of GEM-induced cell death in the IKBKE-overexpressing group (Fig. S2A and S2B). This finding suggests that the modulation of PDAC cell sensitivity to GEM by IKBKE is not markedly associated with AKT/GSK-3β activation. Signaling pathway rewiring and cell death pathway modification are two important strategies that are crucial to cancer treatment sensitivity [31]. AKT/GSK-3β pathway activation is independent of GEM sensitivity. Therefore, we hypothesized that IKBKE influences GEM sensitivity and is associated with the regulation of the death pathway. PDAC cells with downregulated IKBKE were treated with GEM combined with multiple cell death inhibitors, encompassing the autophagy inhibitor chloroquine, ferroptosis inhibitor ferrostatin-1, necroptosis inhibitor necrostatin-1, pyroptosis inhibitor MCC950, apoptosis inhibitor Z-VAD-FMK, caspase-3 inhibitor Z-DEVD-FMK, and GSDMD inhibitor disulfiram. Crystal violet staining (Fig. 5A and B) along with CCK-8 assays (Fig. 5C) were exploited to determine cell viability, with the findings suggesting that MCC950, Z-DEVD-FMK, and Z-VAD-FMK markedly rescued GEM-induced inhibition of cell viability, indicating that IKBKE affects GEM sensitivity in PDAC cells via apoptosis and pyroptosis.

In pancreatic cancer, GEM has been shown to cause pyroptosis and apoptosis [32, 33]. Under GEM stimulation, the typical features of apoptosis and pyroptosis were observed via transmission electron microscopy (Fig. 5D). These features included chromatin condensation, cell shrinkage, and development of apoptotic bodies (indicating apoptosis), as well as marked swelling of organelles, for example the endoplasmic reticulum and mitochondria, accompanied by the formation of bubble-like protrusions on the cell surface (characteristic of pyroptosis). Therefore, more research is needed to determine whether pyroptosis is a key factor in the increased GEM sensitivity resulting from IKBKE knockdown. The percentage of pyroptotic cells of IKBKE-downregulated PDAC cells after GEM therapy was measured by flow cytometry (Fig. 5E–G). The findings indicated that the proportion of pyroptotic cells (Annexin V+, PI+) significantly increased, suggesting increased pyroptosis [34]. Furthermore, cell morphology was observed under a microscope after GEM treatment (Fig. 5H). More cells with bubbly protrusions, a typical morphological characteristic of pyroptosis under microscopy, were detected in the IKBKE-knockdown group. In addition, the result of the LDH release assay indicated more severe cell membrane rupture and leakage of LDH in IKBKE-downregulated PDAC cells following GEM treatment (Fig. 5I). These results implied that IKBKE downregulation enhanced GEM-induced pyroptosis in PDAC cells and that pyroptosis significantly contributed to the increased GEM sensitivity induced by IKBKE knockdown.

### IKBKE downregulation promotes GEM-induced activation of the caspase-3/GSDME pathway in PDAC

**Fig. 6:**
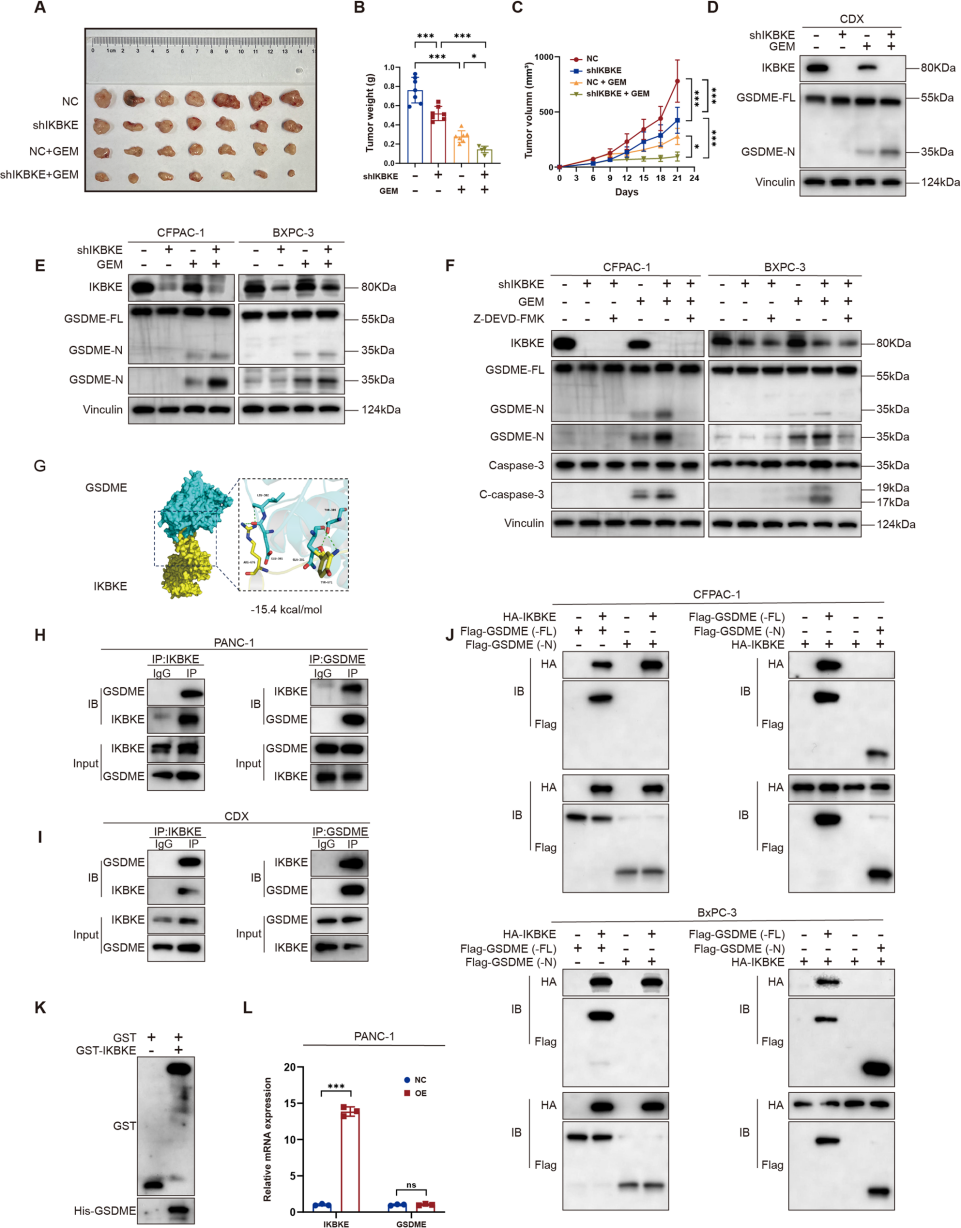
IKBKE downregulation promotes GEM-induced activation of the caspase-3/GSDME pathway in PDAC. **A** Representative photo of excised tumors from xenograft models. Groups: NC (*n* = 7), shIKBKE (*n* = 7), NC+GEM (*n* = 7), shIKBKE+GEM (*n* = 7). **B, C** Quantification of tumor weights (**B**) and volumes (**C**). **D, E** Western blot analysis shows the expression of GSDME (GSDME-FL) and cleaved GSDME (GSDME-N) in IKBKE-knockdown xenograft tumors (**D**) and corresponding cells (**E**) after GEM treatment. **F** GSDME-FL, GSDME-N, caspase-3, and cleaved caspase-3 (C-caspase-3) expression in IKBKE-knockdown cells treated with GEM and Z-DEVD-FMK. **G** Structure-based protein interaction interface analysis between IKBKE (yellow) and GSDME (blue). **H, I** Co-IP experiments showed that IKBKE and GSDME interacted at the protein level in PANC-1 cells (**H**) and xenograft tumor models (**I**). **J** Co-IP experiments showed that IKBKE and GSDME-FL, not GSDME-N, interacted at the protein level. **K** GST pull-down assay showed that IKBKE and GSDME interacted directly. **L** mRNA expression of IKBKE and GSDME in NC and IKBKE-overexpressing cells (*n* = 3). Mean ± SD. *P < 0.05, **P < 0.01, ***P < 0.001.

Our results demonstrated that IKBKE downregulation enhanced GEM-induced pyroptosis (Fig. 5). To further investigate the in vivo regulatory role and translational potential of IKBKE in modulating GEM sensitivity, we established PDAC xenograft tumor models. The results showed that inhibiting IKBKE in vivo significantly potentiated the therapeutic efficacy of GEM, effectively suppressing tumor growth and reducing tumor mass (Fig. 6A –C). Given the recent report by Li et al. [34] that GEM induces pyroptosis via the caspase-3/GSDME pathway, and considering that GSDME is a key executor of pyroptosis, we next investigated whether GSDME mediated the enhanced antitumor effect observed upon IKBKE inhibition. Western blot analysis of GSDME activation in cells (Fig. 6E) and xenograft tumors (Fig. 6D) showed that IKBKE downregulation promoted GSDME cleavage. Moreover, GEM-induced GSDME cleavage was successfully prevented by suppressing caspase-3 activity with the specific inhibitor Z-DEVD-FMK (Fig. 6F). These findings reveal that IKBKE modulates GEM-induced pyroptosis in PDAC cells through the caspase-3/GSDME pathway.

To determine whether an interaction exists between IKBKE and GSDME, we first used molecular docking software to predict the binding energy between the two. This software predicts intermolecular binding affinity using the 3D structures of proteins. The results revealed a binding energy of −15.4 kcal/mol between IKBKE and GSDME (Fig. 6G), indicating a strong interaction [35]. We demonstrated the interaction between endogenous GSDME and IKBKE in PANC-1 cells (Fig. 6H), which was further validated in vivo (Fig. 6I). Next, we used Tet-on plasmids to transfect GSDME-FL or its GSDME-N into BxPC-3 and CFPAC-1 cells (Fig. 6J). The findings exhibited that IKBKE interacted only with GSDME-FL. Furthermore, a direct interaction between GSDME and IKBKE was further verified by GST pull-down assays (Fig. 6K). Collectively, these results demonstrate a physical interaction between IKBKE and GSDME. In addition, IKBKE knockdown did not influence the GSDME mRNA expression levels in PDAC cells (Fig. 6L), suggesting that IKBKE interacts with GSDME at the posttranscriptional level.

### IKBKE inhibits caspase-3–mediated GSDME cleavage through phosphorylation at Thr6

**Fig. 7:**
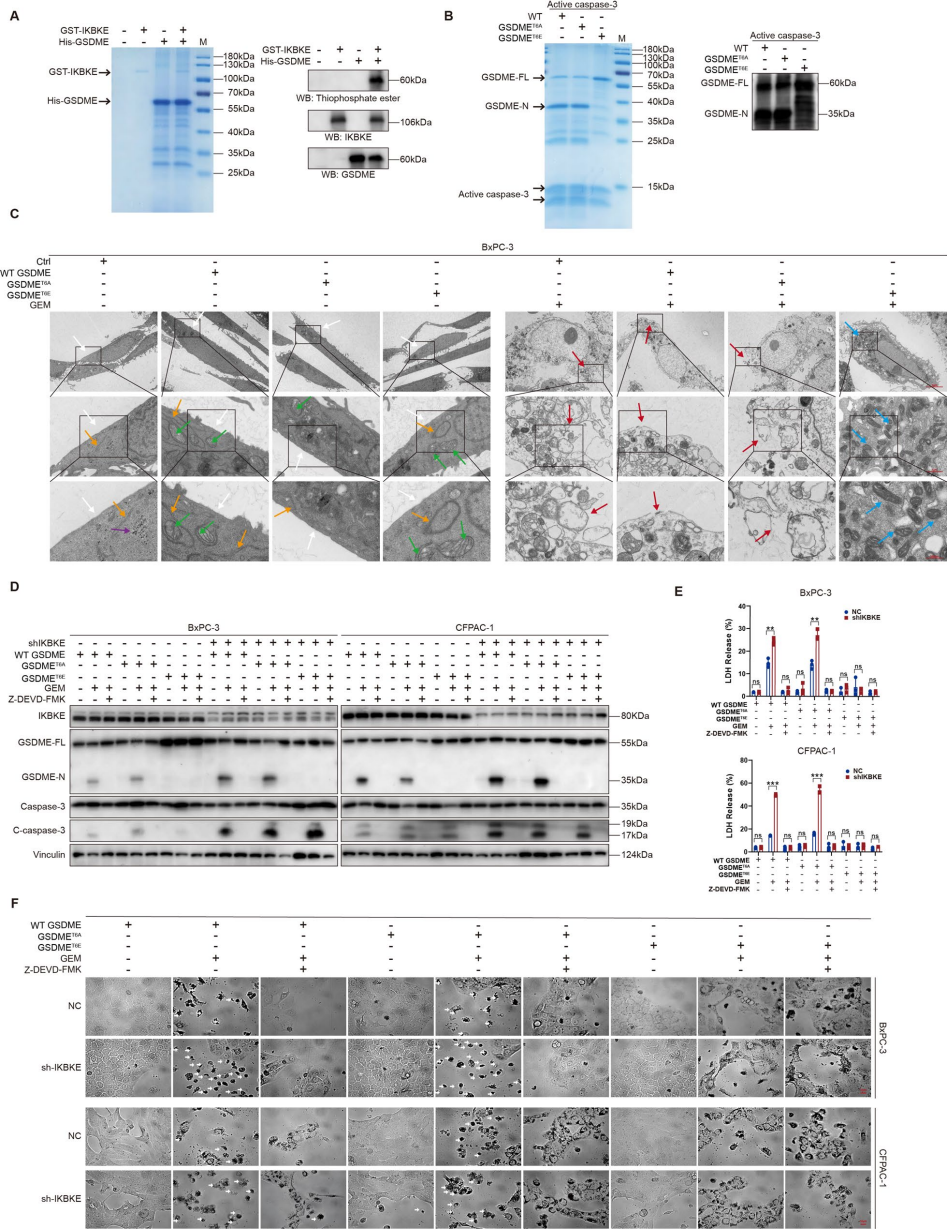
IKBKE inhibits caspase-3–mediated GSDME cleavage through phosphorylation at Thr6. **A** In vitro kinase assay of GST-IKBKE and His-GSDME. **B** Cleavage of human WT GSDME, GSDME^T6A^, and GSDME^T6E^ by the p17/19 active fragments of caspases-3. **C** Transmission electron microscopy showing pyroptotic morphology in BxPC-3 cells transfected with WT GSDME, GSDME^T6A^, and GSDME^T6E^ following GEM treatment. Representative pyroptotic images of BxPC-3 cells via transmission electron microscopy; scale bars = 5 μm, 1 μm and 500 nm, respectively. Arrows indicate the following cellular structures and features: white, intact plasma membrane; green, mitochondria; yellow, endoplasmic reticulum; purple, glycogen granules; red, content leakage at sites of plasma membrane pores; blue, largely preserved organelle architecture. **D** Western blot analysis showing GSDME and caspase-3 cleavage in BxPC-3 and CFPAC-1 cells transfected with WT GSDME, GSDME^T6A^, and GSDME^T6E^ after treatment with GEM and Z-DEVD-FMK. **E** LDH release assays (*n* = 3) in BxPC-3 and CFPAC-1 cells transfected with WT GSDME, GSDME^T6A^, and GSDME^T6E^ following treatment with GEM and Z-DEVD-FMK. **F** Representative light microscopy images of BxPC-3 and CFPAC-1 cells transfected with WT GSDME, GSDME^T6A^, and GSDME^T6E^ after treatment with GEM and Z-DEVD-FMK. White arrows indicate pyroptotic cells with characteristic bubble-like protrusions; scale bar = 20 μm. Mean ± SD. *P < 0.05, **P < 0.01, ***P < 0.001.

Previous studies have reported that GSDME possesses a potential phosphorylation site at Thr6 and that phosphorylation at this site can regulate caspase-3–mediated cleavage [36]. Based on this interaction and the kinase property of IKBKE, we hypothesized that IKBKE regulates GSDME via phosphorylation. In vitro phosphorylation assays supported this hypothesis (Fig. 7A), showing that IKBKE directly phosphorylates GSDME. Mass spectrometry analysis showed that IKBKE specifically phosphorylated GSDME at Thr6 (Fig. S3A). In vitro cleavage assays demonstrated that active caspase-3 cleaved WT GSDME and the nonphosphorylatable T6A (Thr6-to-Ala) mutant, but not the cleavage-resistant phospho-mimetic T6E (Thr6-to-Glu) mutant (Fig. 7B). Transmission electron microscopy further revealed that GEM treatment induced membrane perforation in cells expressing WT GSDME or the T6A mutant, whereas cells expressing the T6E mutant maintained intact plasma membranes (Fig. 7C). These findings indicate that Thr6 phosphorylation of GSDME is essential for IKBKE-mediated inhibition of pyroptosis.

We generated GSDME-knockout cells using CRISPR/Cas9 in the BxPC-3 and CFPAC-1 cell lines. In GSDME-knockout cells, when compared to cells transfected with the WT GSDME plasmid, GEM did not substantially cause pyroptosis or cleavage in GSDME^T6E^-transfected cells, but it did significantly cause GSDME pyroptosis and cleavage in GSDME^T6A^-transfected cells. Besides, cleavage of WT GSDME and GSDME^T6A^ was successfully suppressed by caspase-3 inhibitor Z-DEVD-FMK treatment (Fig. 7D). Under GEM stimulation, cells expressing either WT GSDME or GSDME^T6A^ exhibited increased LDH release (Fig. 7E) and typical bubble-like morphology (Fig. 7F). This effect was completely blocked by Z-DEVD-FMK, confirming its dependence on caspase-3 activation. These results collectively demonstrate that IKBKE inhibits GSDME-dependent pyroptosis by phosphorylating GSDME at Thr6, thereby preventing caspase-3–mediated cleavage.

## Discussion

PDAC is a very aggressive cancer that has a bad prognosis. Although surgery and chemotherapy are available, the overall response rate to chemotherapy in PDAC remains only 10%–20% [37, 38], and the majority of patients derive minimal clinical benefit. These results emphasize the critical need to clarify the processes causing chemoresistance and develop strategies that improve chemosensitivity.

Consistent with previous reports identifying IKBKE as an important oncogenic driver in pancreatic tumorigenesis [39–41], this study further demonstrates that its high expression in PDAC is clinically relevant, being correlated with advanced T stage and poor OS. Functional tests conducted both in vivo and in vitro further showed that IKBKE stimulates PDAC cell migration and proliferation, partly through activation of the AKT/GSK-3β signaling pathway. However, examination of its effect on GEM sensitivity showed that modulation of the AKT/GSK-3β pathway alone was insufficient to account for the impact of IKBKE on chemotherapy, suggesting that alternative mechanisms contribute to IKBKE-mediated regulation of chemoresistance.

IKBKE is a serine/threonine kinase with the primary function of regulating multiple signaling pathways via downstream substrate phosphorylation [42, 43]. Existing studies have reported that Snail can be directly phosphorylated and stabilized by abnormal IKBKE activation or expression, which can lead to Snail-mediated EMT-associated malignant development and contribute to the metastasis of breast cancer [44]. However, it remains unclear whether it directly regulates cell death executor proteins via phosphorylation, thereby influencing chemosensitivity.

Pyroptosis represents an important form of chemotherapy-induced cell death. GEM is one of the chemotherapeutic drugs that can activate caspase-3, which cleaves GSDME to generate GSDME-N, creating membrane holes and triggering pyroptosis [27, 34]. Regulation of this process, particularly through posttranslational modifications such as phosphorylation, can markedly influence chemotherapeutic efficacy and tumor cell fate [36].

Notably, this study demonstrates a direct regulatory role of IKBKE in GSDME-dependent pyroptosis. It shows that IKBKE binds to GSDME and phosphorylates it at the Thr6 residue, thereby preventing caspase-3–mediated cleavage and suppressing GEM-induced pyroptosis. This mechanism is supported by numerous lines of evidence. First, the direct interaction between GSDME and IKBKE was verified by co-IP and GST pull-down assays. Meanwhile, in vitro phosphorylation assays identified Thr6 as a key phosphorylation site. Functional mutagenesis experiments showed that the phospho-mimetic mutant (T6E) markedly inhibited GEM-induced pyroptosis, whereas the nonphosphorylatable mutant (T6A) restored sensitivity to GEM-induced pyroptosis. These findings indicate a novel posttranslational regulatory mechanism underlying IKBKE-mediated modulation of chemotherapeutic sensitivity.

From a therapeutic standpoint, pharmacological IKBKE inhibition using AMX not only suppressed PDAC cell proliferation and migration but also enhanced GEM-induced pyroptosis, thereby restoring chemosensitivity. These findings underscore the therapeutic potential of AMX for PDAC. This result indicates that IKBKE inhibition can serve as an effective strategy for overcoming chemoresistance, particularly in patients with PDAC exhibiting high IKBKE expression.

This study has the following limitations. First, although our experiments confirmed that GSDME Thr6 is the key phosphorylation site through which IKBKE regulates pyroptosis, other potential regulatory mechanisms remain to be further explored. Second, pharmacological inhibition of IKBKE was primarily evaluated in immunodeficient mouse models, which limits a comprehensive assessment of the role of pyroptosis within an intact immune microenvironment. Addressing these limitations will be crucial for translating these findings into clinical practice.

In contrast to the immunosuppressive tumor microenvironment induced by apoptosis, pyroptosis can release a large number of damage-related molecular patterns, thereby effectively activating anti-tumor immune responses [45]. Building on this mechanism and considering the limitations above, future research should focus on: investigating whether the combination of IKBKE inhibitors and chemotherapy can enhance therapeutic efficacy through direct induction of tumor cell death and suppression of malignant phenotypes; systematically evaluating whether this combination strategy can further improve overall treatment outcomes via pyroptosis-related immunomodulatory synergy in immunocompetent models; and comprehensively validating its safety profile and clinical applicability.

In conclusion, our results show that IKBKE is significantly expressed in PDAC and promotes migration and proliferation through the AKT/GSK-3β pathway, which aids in the development of tumors. In order to suppress caspase-3–mediated cleavage, IKBKE phosphorylates GSDME at Thr6, thus inhibiting GEM-induced pyroptosis and conferring chemoresistance. GEM sensitivity is increased by pharmacological IKBKE suppression, suggesting IKBKE as a possible therapeutic target for enhancing PDAC therapy results.

## Supplementary Information

Supplementary Material 1.

Supplementary Material 2.

## Data Availability

This published paper and its supplemental information files contain all of the data produced or examined during this work.

## Abbreviations

AKT: Protein kinase B
AMX: Amlexanox
CCK-8: Cell counting kit-8
CI: Confidence interval
Co-IP: Co-immunoprecipitation
GEM: Gemcitabine
GSDME: Gasdermin E
GSK: Glycogen synthase kinase
GST: Glutathione S-transferase
H&E: Hematoxylin and eosin
HR: Hazard ratio
IHC: Immunohistochemistry
IKBKE: Inhibitor of nuclear factor kappa-B kinase subunit epsilon
IKK: IκB kinase
LDH: Lactate dehydrogenase
NC: Negative control
OE: Overexpression
OS: Overall survival
PAGE: Polyacrylamide gel electrophoresis
PBS: Phosphate-buffered saline
PDAC: Pancreatic ductal adenocarcinoma
PDO: Patient-derived tumor organoid
SD: Standard deviation
SDS: Sodium dodecyl sulfate
