# Supplementary material for: IKBKE downregulation increases chemosensitivity through pyroptosis mediated by the caspase-3/GSDME pathway in pancreatic cancer
Source: J Exp Clin Cancer Res. 2026 Feb 16;45:75. doi: 10.1186/s13046-026-03670-1 (PMC13020273; doi:10.1186/s13046-026-03670-1)
Supplement: Supplementary file 1 — Supplementary Material 1. [file 13046_2026_3670_MOESM1_ESM.docx]

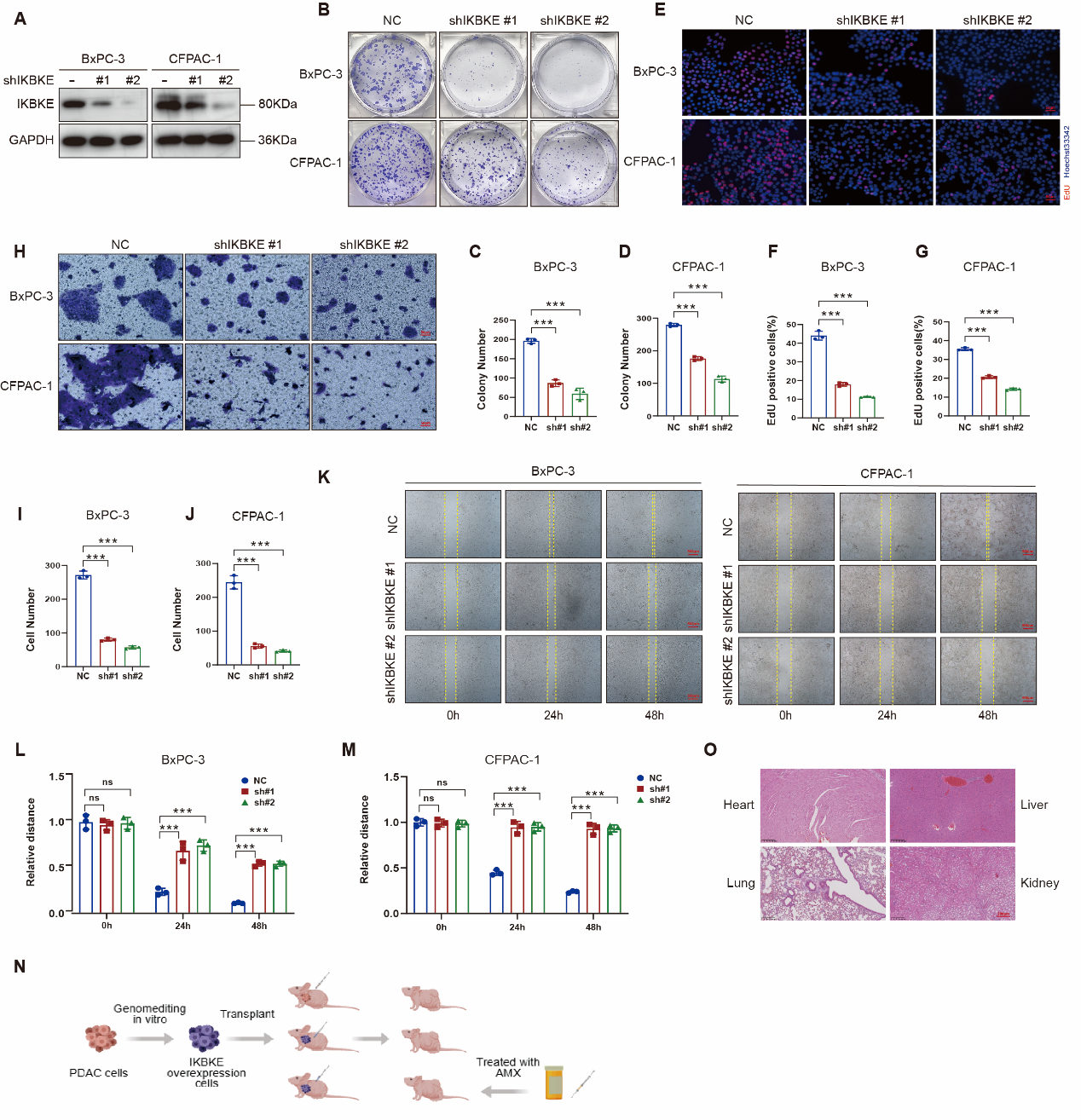


**Fig. S1 IKBKE facilitates the malignant biological behavior of PDAC both *in vitro* and *in vivo*.**

**A** IKBKE expression in stable cell lines transfected with lentivirus encoding shRNA against IKBKE (sh#1 and sh#2).

**B-D** Colony formation assay of IKBKE-knockdown cells (n = 3). Representative images (B) and quantitative analysis of colony numbers (C, D) are shown.

**E-G** Effect of IKBKE on DNA synthesis in IKBKE-knockdown cells (n = 3). Cells were fluorescently stained with EdU (red). The nucleus was stained with Hoechst 33342 (blue); scale bar = 50 μm. Representative images (E) and quantification of the EdU-positive cells (F, G) are shown.

**H-J** Images and quantification of Transwell migration for IKBKE-knockdown cells (n = 3). (H) A representative field (scale bar = 50 μm). (I, J) Number of migrated cells per field.

**K-M** Wound healing assay of IKBKE-knockdown cells (n = 3); scale bar = 500 μm. Representative pictures (K) and quantifications (L, M) are shown.

**N** Illustration of the methodology used to establish PDAC xenograft tumor models.

**O** H&E pathologic sections of the heart, liver, kidney, and lungs in BALB/c nude mice; scale bar = 200 μm.

Mean ± SD. **P* <0.05, ***P* <0.01, ****P* <0.001


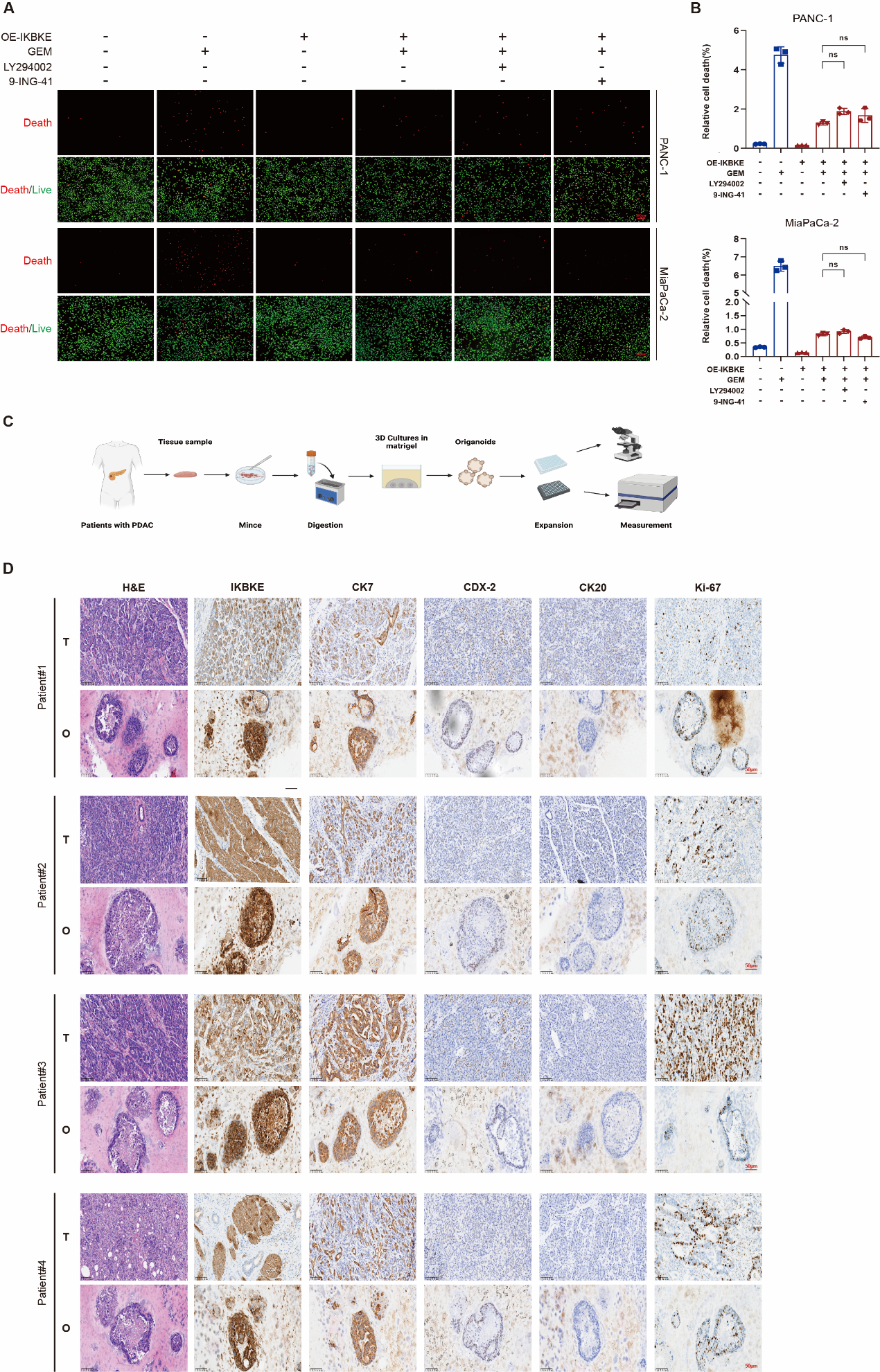


**Fig. S2 IKBKE affects PDAC sensitivity to GEM.**

**A, B** Live/dead cell assay (n = 3) in IKBKE-overexpressing cells. Cells were treated with GEM, the AKT inhibitor LY294002, or the GSK-3β inhibitor 9-ING-41. Dead cells are shown in red and live cells in green; scale bar = 200 μm. The right panel quantifies the relative cell death (B).

**C** Schematic illustration of patient-derived organoid (PDO) development (created with BioRender.com).

**D** Histopathological characterization of PDOs. Representative images of H&E staining and immunohistochemistry (IHC) for IKBKE, CK7, CDX2, CK20, and Ki-67 are shown; scale bar = 50 μm.

Mean ± SD. **P* <0.05, ***P* <0.01, ****P* <0.001


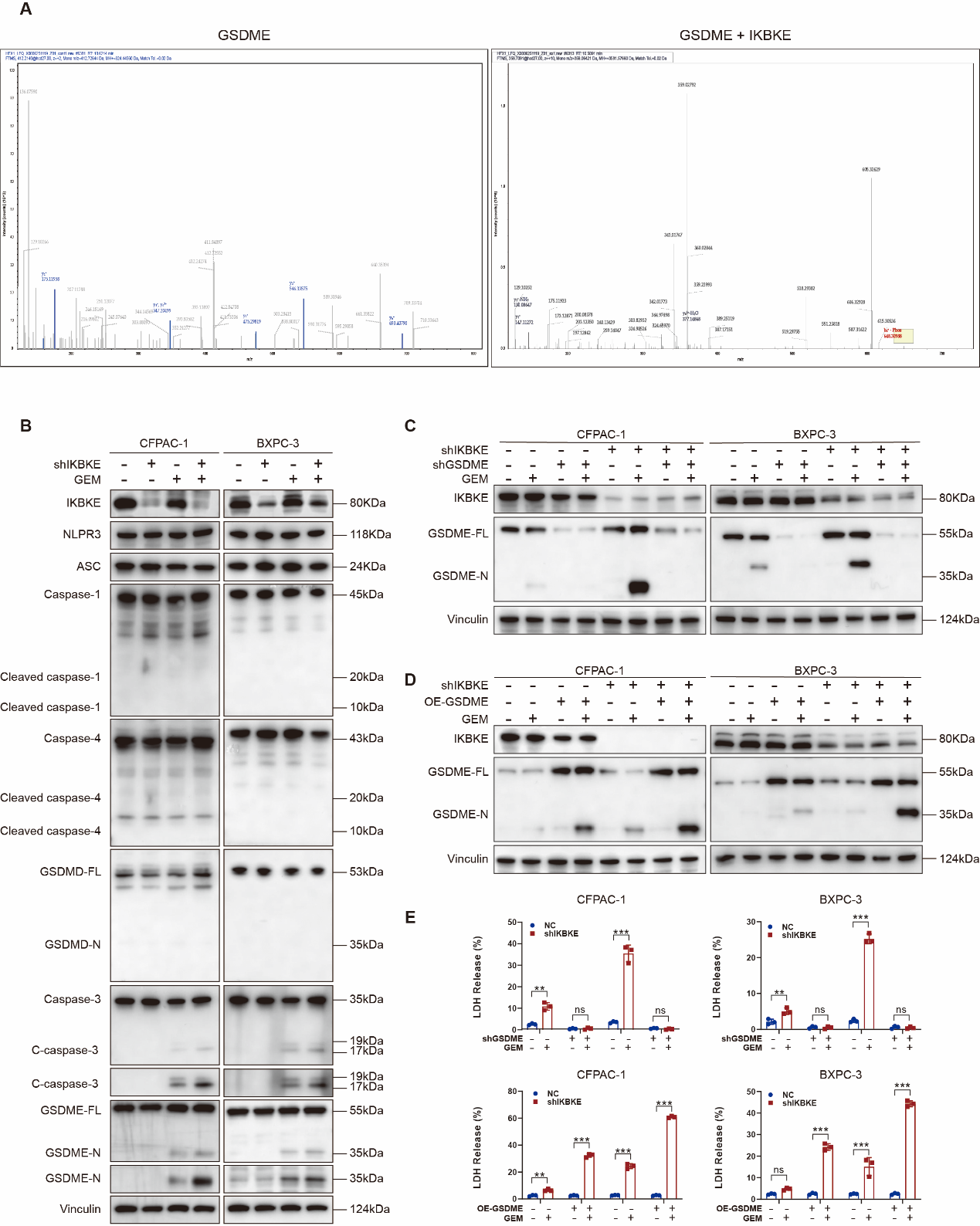


**Fig. S3**

**A** Mass spectrometric analysis of *in vitro* kinase assay products. The reaction mixture containing GST-IKBKE and His-GSDME was analyzed to identify phosphorylation sites.

**B** Analysis of pyroptosis-related proteins by Western blot. The expression of key proteins involved in pyroptosis was examined.

**C, D** GSDME cleavage in response to GSDME modulation and GEM treatment. Western blot analysis of GSDME-FL and cleaved GSDME (GSDME-N) expression in BxPC-3 and CFPAC-1 cells transfected with shGSDME (C) or GSDME overexpression (OE-GSDME) (D) constructs.

**E** LDH release assay (n = 3). BxPC-3 and CFPAC-1 cells, transfected as indicated and treated with GEM, were assessed for pyroptosis-related membrane rupture by measuring LDH release.

Mean ± SD. **P* <0.05, ***P* <0.01, ****P* <0.001
