# Supplementary material for: IKBKE downregulation increases chemosensitivity through pyroptosis mediated by the caspase-3/GSDME pathway in pancreatic cancer
Source: J Exp Clin Cancer Res. 2026 Feb 16;45:75. doi: 10.1186/s13046-026-03670-1 (PMC13020273; doi:10.1186/s13046-026-03670-1)
Supplement: Supplementary file 2 — Supplementary Material 2. [file 13046_2026_3670_MOESM2_ESM.docx]

**Supplementary Table S1.**

| **Clinicopathological characteristics of the 103 PDAC patients** | | |
| --- | --- | --- |
| **Characteristic** | **Patients, n** | **Percentage (%)** |
| **Age** |  |  |
| <59 | 50 | 48.5 |
| ≥59 | 53 | 51.5 |
| **Gender** |  |  |
| Female | 34 | 33.0 |
| Male | 69 | 67.0 |
| **Location** |  |  |
| Head of pancreas | 47 | 45.6 |
| Body and tail of pancreas | 56 | 54.4 |
| **Histologic grade** |  |  |
| G1 | 8 | 7.8 |
| G2 | 57 | 55.3 |
| G3 | 38 | 36.9 |
| **T stage** |  |  |
| T1 | 27 | 26.2 |
| T2 | 42 | 40.8 |
| T3 | 26 | 25.2 |
| T4 | 8 | 7.8 |
| **N stage** |  |  |
| N0 | 63 | 61.2 |
| N1 | 34 | 33.0 |
| N2 | 6 | 5.8 |
| **AJCC stage** |  |  |
| Ⅰ | 52 | 50.5 |
| Ⅱ | 43 | 41.7 |
| Ⅲ | 8 | 7.8 |
| **Short-term death (≤ 6 months)** |  |  |
| Yes | 27 | 26.2 |
| No | 76 | 73.8 |
| **Adjuvant therapy** |  |  |
| Yes | 13 | 12.6 |
| No | 90 | 87.4 |
| **Hypertension** |  |  |
| Yes | 25 | 24.3 |
| No | 78 | 75.7 |
| **Diabetes** |  |  |
| Yes | 25 | 24.3 |
| No | 78 | 75.7 |
| **Coronary heart disease** |  |  |
| Yes | 5 | 4.8 |
| No | 98 | 95.2 |
| **Second primary tumor** |  |  |
| Yes | 3 | 2.9 |
| No | 100 | 97.1 |
| **Comorbidity (hypertension, diabetes, coronary heart disease, second primary tumor)** |  |  |
| Yes | 41 | 39.8 |
| No | 62 | 60.2 |
